# Supplementary material for: German Translation and Cross-Cultural Adaptation of the Limb Deformity-Scoliosis Research Society (LD-SRS) Questionnaire
Source: Healthcare (Basel). 2022 Jul 13;10(7):1299. doi: 10.3390/healthcare10071299 (PMC9322954; doi:10.3390/healthcare10071299)
Supplement: Supplementary file 1 [file healthcare-10-01299-s001.zip › Table S1 LD-SRS German without scoring instructions(for patients).pdf]

LD-SRS 30

Ihre behandelnden Ärzte möchten sorgfältig den Zustand Ihrer Gliedmaße vor und nach Ihrer Behandlung untersuchen.
Bitte kreuzen Sie die zutreffendste Antwort an.
Wenn Sie schon operiert worden sind, bitte füllen Sie die Teil 1 und 2 aus.
Wenn Sie noch nicht operiert worden sind, dann nur Abschnitt 1 ausfüllen. **Danke!**

Alle Angaben werden vertraulich behandelt.

Name: ______________________________

Geschlecht: ______________________________

Geburtsdatum: ______________________________

Heutiges Datum: ______________________________

Operationsdatum / Behandlungsbeginn: ______________________________

In dem folgenden Fragebogen bezieht sich der Begriff "Gliedmaße" auf das Körperteil, das behandelt wird, inklusive des Gelenks darüber und darunter. Falls mehr als ein Körperteil behandelt wird, füllen Sie bitte jeweils einen separaten Fragebogen aus.

Körperteil

- ☐ Oberarm
- ☐ Unterarm
- ☐ Hand/Handgelenk
- ☐ Oberschenkel
- ☐ Unterschenkel
- ☐ Fuß/Sprunggelenk

Seite

- ☐ Rechts
- ☐ Links

Teil 1: Alle Patienten

1. **Mit welcher der folgenden Antworten lassen sich die Schmerzen, die Sie in den vergangenen 6 Monaten erlitten haben, am besten beschreiben?**
   - ☐ keine
   - ☐ leichte
   - ☐ mäßige
   - ☐ mäßige bis starke
   - ☐ starke

2. **Mit welcher der folgenden Antworten lassen sich die Schmerzen, die Sie im vergangenen Monat erlitten haben, am besten beschreiben?**
   - ☐ keine
   - ☐ leichte
   - ☐ mäßige
   - ☐ mäßige bis starke
   - ☐ starke

3. **Waren Sie in den vergangenen 6 Monaten sehr nervös?**
   - ☐ zu keinem Zeitpunkt
   - ☐ für kurze Zeit
   - ☐ einige Zeit
   - ☐ die meiste Zeit
   - ☐ die ganze Zeit

4. **Wie würden Sie sich dabei fühlen, wenn Sie Ihr restliches Leben mit der momentanen Form Ihrer Gliedmaße verbringen müssten?**
   - ☐ sehr glücklich
   - ☐ ziemlich glücklich
   - ☐ weder glücklich noch unglücklich
   - ☐ ziemlich unglücklich
   - ☐ sehr unglücklich

5. **Auf welcher Ebene bewegen sich Ihre Aktivitäten gegenwärtig?**
   - ☐ sämtliche Aktivitäten ohne Einschränkungen
   - ☐ mäßige körperliche Arbeit und mäßige sportliche Betätigung, wie z.B. Wandern und Radfahren
   - ☐ leichte Arbeit, wie z.B. Hausarbeit
   - ☐ im Wesentlichen keine Aktivität
   - ☐ bettlägerig / Rollstuhl

6. **Wie sehen Sie in Kleidung aus?**
   - ☐ sehr gut
   - ☐ gut
   - ☐ einigermaßen gut
   - ☐ schlecht
   - ☐ sehr schlecht

7. Wie häufig haben Sie sich in den vergangen 6 Monaten so niedergeschlagen gefühlt, dass nichts Sie aufheitern konnte?

- ☐ sehr häufig
- ☐ häufig
- ☐ gelegentlich
- ☐ selten
- ☐ nie

8. Haben Sie Schmerzen in Ihrer Gliedmaße in Ruhe?

- ☐ sehr häufig
- ☐ häufig
- ☐ gelegentlich
- ☐ selten
- ☐ nie

9. Auf welcher Ebene bewegen sich gegenwärtig Ihre beruflichen/schulischen Aktivitäten?

- ☐ 100% normal
- ☐ 75% normal
- ☐ 50% normal
- ☐ 25% normal
- ☐ 0% normal

10. Mit welcher der folgenden Antworten lässt sich am besten das Aussehen Ihrer Gliedmaße beschreiben?

- ☐ sehr gut
- ☐ gut
- ☐ einigermaßen gut
- ☐ schlecht
- ☐ sehr schlecht

11a. Was für Medikamente nehmen Sie aufgrund Ihrer Beschwerden an der Gliedmaße ein?

Bitte nur eine Antwort wählen)

- ☐ keine
- ☐ Nichtbetäubungsmittel wöchentlich oder weniger (z.B. Paracetamol, Ibuprofen)
- ☐ Nichtbetäubungsmittel täglich
- ☐ Betäubungsmittel wöchentlich oder weniger (z.B. Oxycodon, Hydrocodon, Codein, Propoxyphen)
- ☐ Betäubungsmittel täglich
- ☐ Sonstige (bitte unten angeben)

b. “sonstige”, spezifizieren Sie Medikament:

____

c. „sonstige“, spezifizieren Sie Ihre Verwendung: täglich/ wöchentlich oder weniger

____

12. Schränkt Ihre Gliedmaße Ihre Fähigkeiten ein, zu Hause etwas zu tun?

- ☐ nie
- ☐ selten
- ☐ gelegentlich
- ☐ häufig
- ☐ sehr häufig

13. Waren Sie in den vergangenen 6 Monaten ruhig und gelassen?

- ☐ die ganze Zeit
- ☐ die meiste Zeit
- ☐ einige Zeit
- ☐ für kurze Zeit
- ☐ zu keinem Zeitpunkt

14. Sind Sie der Meinung, dass sich die Erkrankung Ihrer Gliedmaße auf Ihre persönlichen Beziehungen auswirkt?

- ☐ überhaupt nicht
- ☐ kaum
- ☐ schwach
- ☐ mäßig
- ☐ stark

15. Haben Sie und/oder Ihre Familie wegen Ihrer Gliedmaße "finanzielle Schwierigkeiten?

- ☐ starke
- ☐ mäßige
- ☐ leichte
- ☐ kaum
- ☐ überhaupt keine

16. Waren Sie in den vergangenen 6 Monaten niedergeschlagen und traurig?

- ☐ nie
- ☐ selten
- ☐ gelegentlich
- ☐ häufig
- ☐ sehr häufig

17. Haben Sie sich in den vergangenen 3 Monaten bei der Arbeit bzw. in der Schule aufgrund von Schmerzen in Ihrer Gliedmaße krank gemeldet? Falls ja, wie viele Tage?

- ☐ 0 Tage
- ☐ 1 Tag
- ☐ 2 Tage
- ☐ 3 Tage
- ☐ 4 oder mehr Tage

18. Gehen Sie häufiger oder seltener aus als Ihre Freunde?

- ☐ viel häufiger
- ☐ häufiger
- ☐ gleich viel
- ☐ seltener
- ☐ viel seltener

19. Halten Sie sich mit dem gegenwärtigen Zustand Ihrer Gliedmaße für attraktiv?

- ☐ ja, sehr
- ☐ ja, ziemlich
- ☐ weder attraktiv noch unattraktiv
- ☐ nein, nicht sehr
- ☐ nein, überhaupt nicht

20. Waren Sie in den vergangenen 6 Monaten glücklich?

- ☐ zu keinem Zeitpunkt
- ☐ für kurze Zeit
- ☐ einige Zeit
- ☐ die meiste Zeit
- ☐ die ganze Zeit

21. Sind Sie mit den Ergebnissen der Behandlung Ihrer Gliedmaße zufrieden?

- ☐ sehr zufrieden
- ☐ zufrieden
- ☐ weder zufrieden noch unzufrieden
- ☐ unzufrieden
- ☐ sehr unzufrieden

22. Würden Sie sich noch einmal derselben Behandlung unterziehen, wenn Sie an denselben
Beschwerden litten?

- ☐ ja, bestimmt
- ☐ wahrscheinlich ja
- ☐ bin mir nicht sicher
- ☐ wahrscheinlich nicht
- ☐ nein, bestimmt nicht

23. Wie würden Sie Ihr Selbstbild auf einer Skala von 1 bis 9 bewerten, auf der 1 der schlechteste und 9 der beste Wert ist?

☐ 1 ☐ 2 ☐ 3 ☐ 4 ☐ 5 ☐ 6 ☐ 7 ☐ 8 ☐ 9

Die folgenden Fragen sind nur zu beantworten, falls Sie die Behandlung Ihrer Gliedmaße schon begonnen haben.
Haben Sie die Behandlung bereits begonnen?

- ☐ ja
- ☐ nein

24. Wie finden Sie Ihr Aussehen jetzt im Vergleich zu vor der Behandlung?

- ☐ viel besser
- ☐ besser
- ☐ gleich
- ☐ schlechter
- ☐ viel schlechter

25. Haben sich durch die Behandlung Ihrer Gliedmaße Ihre Leistungsfähigkeit und Ihre täglichen Aktivitäten verändert?

- ☐ gesteigert
- ☐ nicht verändert
- ☐ vermindert

26. Hat sich durch die Behandlung Ihrer Gliedmaße Ihre Fähigkeit verändert, Sport/Hobbys zu genießen?

- ☐ gesteigert
- ☐ nicht verändert
- ☐ vermindert

27. Wie haben sich die Schmerzen in Ihrer Gliedmaße durch die Behandlung Ihrer Gliedmaße verändert?

- ☐ gesteigert
- ☐ nicht verändert
- ☐ vermindert

28. Hat die Behandlung Ihr Selbstvertrauen hinsichtlich persönlicher Beziehungen mit anderen Menschen verändert?

- ☐ gesteigert
- ☐ nicht verändert
- ☐ vermindert

29. Hat die Behandlung die Art verändert, wie andere Menschen Sie sehen?

- ☐ viel besser
- ☐ besser
- ☐ gleich
- ☐ schlechter
- ☐ viel schlechter

30. Hat die Behandlung Ihr Selbstbild verändert?

- ☐ verbessert
- ☐ nicht verändert
- ☐ verschlechtert

Haben Sie Schmerzen? Falls ja, mit welcher der folgenden Antworten lassen diese sich am besten beschreiben?

- ☐ keine
- ☐ Kribbeln
- ☐ stechender Schmerz
- ☐ brennender Schmerz
- ☐ Tiefenschmerz

Kommentare:
